# Dissection of Cell Death Induction by *Arabidopsis thaliana* CC-NBS-LRR Receptor SUT1 and Its Interacting Protein TOPP4 Mutant in *Nicotiana benthamiana*

**DOI:** 10.3390/life16020227

**Published:** 2026-01-29

**Authors:** Jianzhong Huang, Xiaoqiu Wu, Kai Chen, Zhiyong Gao

**Affiliations:** 1 Department of Basic Medicine, Fuzhou Medical College, Fuzhou 344000, China; ck19851227@163.com; 2 College of Life Sciences, Wuhan University, Wuhan 430072, China; xiaoqiuwuh@163.com; 3 Puai Medical College, Shaoyang University, Shaoyang 422000, China

**Keywords:** *Arabidopsis thaliana*, cell death, *Nicotiana benthamiana*, SUT1, TOPP4

## Abstract

Nucleotide-binding and leucine-rich repeat receptors (NLRs) play an important role in plant innate immunity. Previous reports indicate that SUT1 (SUPPRESSOR OF TOPP4 1) is required for the autoimmune response mediated by TYPE ONE PROTEIN PHOSPHATASE 4 (TOPP4) mutation topp4-1 (namely TOPP4^T246M^) in *Arabidopsis*. We observed that co-expression of SUT1 with TOPP4 mutant versions, instead of wild-type TOPP4, produced robust cell death in *N. benthamiana*. The YFP-labeled SUT1 was localized on the plasma membrane (PM), and Gly2, Cys4, and Ser6 are crucial amino acid sites for its PM localization and function. Further dissection proclaimed that the function and localization of SUT1 are influenced by mutations in conserved specific residues. These findings may provide a new perspective for elucidating the activation mechanism of SUT1.

## 1. Introduction

Plants have developed two layers of immune systems, known as pattern-triggered immunity (PTI) and effector-triggered immunity (ETI), to protect them from the invasion of multifarious pathogens [1,2]. PTI is the fundamental immunity, and its activation depends on surface-localized pattern recognition receptors (PRRs) [3]. Pathogens deploy specific effectors to inhibit PTI [4]. Correspondingly, plants evolved the nucleotide-binding and leucine-rich repeat receptors (NLRs) to recognize the presence of specific effectors to trigger ETI [5]. A typical characteristic of NLR activation is a rapid programmed cell death response, termed hypersensitive response (HR) [6,7].

Plant NLRs can be further subclassified into three categories: CC-NBS-LRR (CNL), TIR-NBS-LRR (TNL), and RPW8-NBS-LRR (RNL) [8,9]. The activation of NLRs can either directly associate with their cognate effectors or indirectly detect the effector-mediated biochemical modifications of plant target proteins (namely ‘guardee’ or ‘decoy’ proteins) [10,11]. The *Arabidopsis* RIN4 is a well-researched guardee protein targeted by multiple effectors, such as AvrRpm1, AvrB, and AvrRpt2 [12]. Two CNL receptors, RPM1 and RPS2, monitor the phosphorylation and cleavage of RIN4 by effectors, respectively [13]. Effectors AvrRpm1 and AvrB directly interact with RIN4 and induce its phosphorylation modification, thereby activating RPM1-mediated defense responses [14]. Effector AvrRpt2, as a protease, directly cleaves RIN4, causing RPS2-triggered immune response [15]. Effector AvrPphB activates ETI by cleaving the *Arabidopsis* PBS1 protein, which is guarded by the CNL receptor RPS5 [16].

Accumulating results confirmed that the subcellular localization of the NLR protein is closely related to its function. *Arabidopsis* RPM1 and RPS5 need plasma membrane (PM) distribution, and disrupting the correct localization of these two NLRs leads to impaired function [17,18]. The RGA4-RGA5 pair in rice is localized in the cytosol [19]. L6 is localized on the Golgi, and R3a requires endomembrane localization [20,21]. MLA10 and Rx exhibit nuclear-cytoplasmic localization, but their activation mechanisms are different. After recognizing cognate effector, MLA10 triggers disease resistance signaling in the nucleus and cell-death signaling in the cytoplasm, while effector-dependent activation of Rx1 occurs only in the cytoplasm [22,23,24]. Different subcellular localization indicates distinct NLR activation mechanisms.

The *Arabidopsis* resistance gene *SUT1* encodes a CNL receptor. Yan et al. reported that SUT1 (SUPPRESSOR OF TOPP4 1) is required for the autoimmune response mediated by TYPE ONE PROTEIN PHOSPHATASE 4 (TOPP4) mutation topp4-1 (namely TOPP4^T246M^) in *Arabidopsis*, and SUT1 physically interacts with TOPP4 and TOPP4^T246M^ in the PM and cytoplasm in *N. benthamiana* leaves. TOPP4^T246M^ activates the autoimmune response outside the nucleus and promotes the accumulation of SUT1 at both the mRNA and the protein levels [25]. Currently, it is unclear whether SUT1 activation can be dissected independently of its transcriptional upregulation and whether membrane localization is directly required for receptor function. *N. benthamiana* serves as an essential model and functional validation platform for studying *Arabidopsis* NLR genes, owing to its rapid transient expression, efficient genetic silencing, evolutionary complementarity with *Arabidopsis*, and high susceptibility to pathogens [26,27,28]. Here, we characterized the induction of cell death by SUT1 in the presence and absence of the TOPP4 mutant using *Agrobacterium*-mediated transient expression in *N. benthamiana*. We observed that co-expression of SUT1 with TOPP4 mutant versions, instead of wild-type TOPP4, produced robust cell death in *N. benthamiana*. YFP-labeled SUT1 was localized on the PM, and the residues Gly2, Cys4, and Ser6 are crucial for its localization and function. Further dissection indicated that the function and localization of SUT1 are influenced by mutations in specific residues. These findings may provide a new perspective for elucidating the activation mechanism of SUT1.

## 2. Materials and Methods

### 2.1. Plant Materials and Growth Conditions

*N. benthamiana* plants were grown in a greenhouse under a 16 h-light/8 h-dark photoperiod at 25 °C. For Agrobacterium-mediated transient expression, we used three leaves from approximately 4-week-old plants, which typically corresponded to the four-leaf stage.

### 2.2. Vectors Construction

cDNA from wild-type *Arabidopsis* (Col-0) leaves served as the initial template. The gel-purified DNA fragments, which encompassed SUT1 and its mutants as well as TOPP4 and its alleles, were individually cloned into a modified pUC19 plasmid containing attL1 and attL2 sites using a one-step cloning kit (Vazyme, Nanjing, China, #C112-02). All constructs were fully sequenced to confirm the absence of unintended nucleotide changes before being transferred into expression vectors via Gateway LR cloning (Thermo Fisher Scientific, Waltham, MA, USA). These vectors included the pEarleygate101 plasmid, which contains attR1 and attR2 sites and was used for protein expression with YFP-HA, 5xMyc, YFP-HA-Rop, or YFP-HA-mRop tags. The final expression plasmids were introduced into *Agrobacterium tumefaciens* GV3101 by electroporation.

### 2.3. Transient Expression Assays in Nicotiana benthamiana

The GV3101 strain containing the specified expression plasmid was grown overnight in liquid LB medium supplemented with kanamycin and rifampicin. The culture was then pelleted and resuspended in an MMS solution (10 mmol MgCl_2_, 10 mmol MES [pH 5.8], 150 μM acetosyringone). This Agrobacterium suspension was infiltrated into the leaves of *N. benthamiana*. For both individual and co-expression assays, the optical density at 600 nm of suspensions for SUT1 and its mutants, as well as for TOPP4 and its alleles, was adjusted to 0.4.

### 2.4. Protein Extraction and Immunoblot Analysis

Three 8 mm leaf disks were harvested, and total protein was extracted using 100 μL of extraction buffer (20 mM Tris-HCl (pH 7.5), 150 mM NaCl, 1 mM EDTA, 1% SDS, and 10 mM DL-dithiothreitol (DTT)). The lysate was boiled at 100 °C for 10 min after adding 1× protein loading buffer. The total protein extract was then centrifuged at 10,000× *g* for 3 min. A 30 μL aliquot of the supernatant was separated by 10% SDS–PAGE gel and transferred to a nitrocellulose membrane for immunoblotting. Primary antibodies used were anti-HA (Roche, Basel, Switzerland, #11867423001), anti-Myc (Genscript, Nanjing, China, #A00704), and anti-β-actin (Abbkine, Wuhan, China, #A01050-2). The substantial molecular weight difference between YFP-HA-labeled SUT1 and its mutants (~120 kDa) and 5xMyc-tagged TOPP4 and its mutants (~42 kDa) or plant β-actin (~43 kDa) allowed the nitrocellulose membrane to be cut appropriately. This enabled the simultaneous detection of both proteins using anti-HA and anti-Myc or anti-β-actin antibodies.

### 2.5. Subcellular Localization Observation

A fluorescence microscope (Olympus BX53, Tokyo, Japan) was used to observe live cells on the abaxial sides of *N. benthamiana* leaves at 28 or 40 h after target protein expression.

## 3. Results

### 3.1. Activation of SUT1 Can Be Driven by TOPP4 Mutants

**Figure 1:**
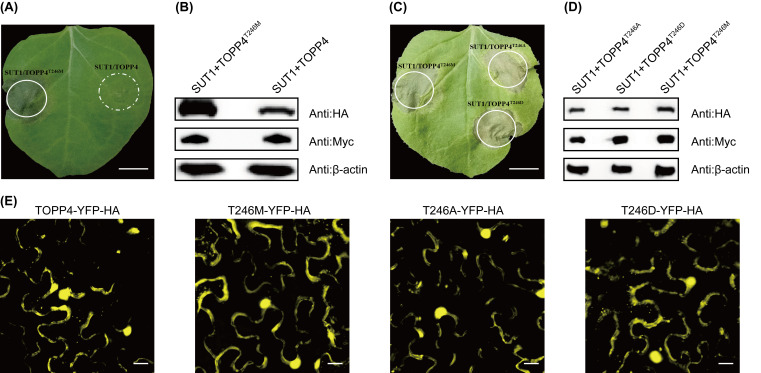
Activation of SUT1 can be driven by TOPP4 mutants. (**A**) Co-expression with TOPP4^T246M^, rather than TOPP4, can activate SUT1 in *Nicotiana benthamiana*. *35S::SUT1-YFP-HA* (OD_600_ = 0.4) was transiently co-expressed with *35S::TOPP4^T246M^-Myc* (OD_600_ = 0.4) or *35::TOPP4-Myc* (OD_600_ = 0.4) in *N. benthamiana* by Agrobacterium infiltration. The picture was photographed at 72 h post-infiltration (hpi). (Scale bar: 1 cm). (**B**) Co-expression with TOPP4^T246M^ increases the protein level of SUT1. Protein samples were extracted at 40 hpi. The protein level of SUT1-YFP-HA, TOPP4^T246M^-Myc, or TOPP4-Myc was detected with anti-HA and anti-Myc antibodies, respectively. β-actin was used as an equal loading of protein. (**C**) Activation of SUT1 can be driven by TOPP4 mutants. (Scale bar: 1 cm). (**D**) Co-expression of SUT1-YFP-HA and Myc-tagged TOPP4 mutants in *N. benthamiana*. (**E**) Observation of the subcellular localization of TOPP4 and TOPP4 alleles with a fluorescence microscope. (Scale bar: 20 μm). TOPP4-YH and TOPP4 alleles-YH were transiently expressed in *N. benthamiana*. Images were observed at 40 hpi. All data are the mean of at least three independent experiments showing consistent results.

We employed an *Agrobacterium*-mediated transient expression system to investigate whether the TOPP4 mutants affect the SUT1-mediated HR in *N. benthamiana*. Single expression of YFP-HA-tagged SUT1 and Myc-tagged TOPP4 or TOPP4^T246M^ did not induce HR (Figure S1A). Co-expression of SUT1 with TOPP4^T246M^, but not TOPP4, induced HR in the leaves of *N. benthamiana,* and co-expression of TOPP4^T246M^ increased the protein level of SUT1 (Figure 1A,B). These results are consistent with previous data from *Arabidopsis* [25], indicating that the transient expression system is suitable to characterize the structure and function of SUT1. Furthermore, the truncated forms (N- or C-terminal) of TOPP4^T246M^ cannot induce the activation of SUT1 (Figure S1B).

Given that RPM1 is activated by monitoring the phosphorylation status of RIN4 at specific sites, such as T166. Phosphomimic mutant of RIN4^T166D^ results in effector-independent RPM1 activation, and RIN4^T166A^ compromises effector-dependent RPM1 activation [14]. We generated TOPP4^T246D^ and TOPP4^T246A^, a phosphomimic and non-phosphomimic mutant of TOPP4, respectively. Neither TOPP4^T246D^ nor TOPP4^T246A^ caused HR independently (Figure S1C). The two mutants function as TOPP4^T246M^ that can cause SUT1-mediated cell death, and they have the same subcellular distribution patterns (Figure 1C–E). These results indicate that SUT1 guards the TOPP4 protein activity, but SUT1 activation is unlikely to be achieved by monitoring the phosphorylation status of TOPP4 at T246.

### 3.2. The Function of SUT1 Is P-Loop Dependent

**Figure 2:**
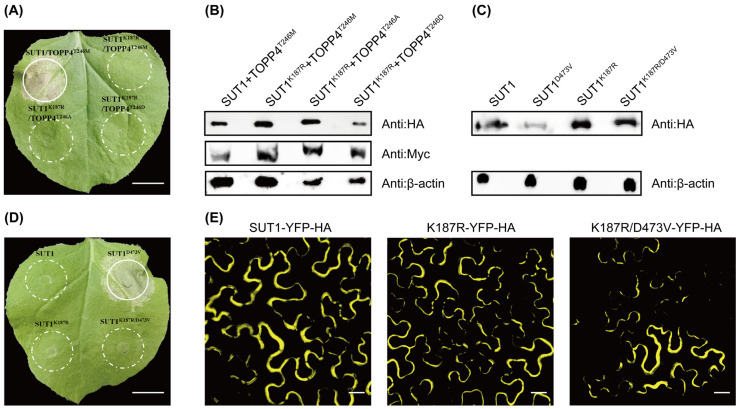
The function, rather than localization, of SUT1 is P-loop-dependent. (**A**) TOPP4 mutants activate SUT1 dependent on the integrity of the P-loop motif. (Scale bar: 1 cm). (**B**) Co-expression of YFP-HA-tagged SUT1^K187R^ and Myc-tagged TOPP4 mutants in *N. benthamiana.* (**C**) Protein levels of the four proteins. Proteins were extracted and detected at 28 hpi before the onset of cell death. (**D**) Analysis of the effect of P-loop and MHD motifs on SUT1. SUT1 and SUT1^D473V^, the MHD mutant, SUT1^K187R^, the P-loop mutant, and the SUT1^K187R/D473V^ double mutant were transiently expressed in *N. benthamiana* (OD_600_ = 0.4). The picture was photographed at 72 hpi. (Scale bar: 1 cm). (**E**) Observation of the subcellular localization of SUT1, SUT1^K187R^, and SUT1^K187R/D473V^ with a fluorescence microscope. (Scale bar: 20 μm). All data represent results from one of at least three independent experiments, which showed consistent results.

The P-loop motif is highly conserved in NBS-LRR proteins [24,29] (Figure S2). We produced a P-loop mutant SUT1^K187R^ to address whether the function of SUT1 requires a wild-type P-loop. TOPP4 versions-mediated activation of SUT1^K187R^ is completely impaired (Figure 2A,B). This result demonstrates that activation of SUT1 by TOPP4 alleles is regulated by canonical P-loop function.

Mutations in the conserved MHD motif can cause autoactivity of NLR proteins, possibly due to intramolecular conformational rearrangement [17,30]. We constructed YFP-HA-epitope-tagged SUT1^D473V^ mutation, and SUT1^D473V^ was sufficient to induce HR. The double mutant SUT1^K187R/D473V^ is functionally deficient. For the MHD mutant SUT1^D473V^, the Western blot shows substantially reduced protein accumulation compared to wild-type SUT1. This likely contributes to the absence of HR. These data suggest that the autoactivation of SUT1^D473V^ mimics P-loop-dependent activation of SUT1 triggered by TOPP4 alleles (Figure 2C,D).

In consistent with previous study, SUT1-YFP-HA is observed predominantly localized on the PM [25,31]. Both SUT1^K187R^-YFP-HA and SUT1^K187R/D473V^-YFP-HA displayed the same subcellular localization as SUT1-YFP-HA (Figure 2E). These results indicate that the PM localization of SUT1 is not P-loop dependent.

### 3.3. PM Localization Is Crucial for the Function of SUT1

**Figure 3:**
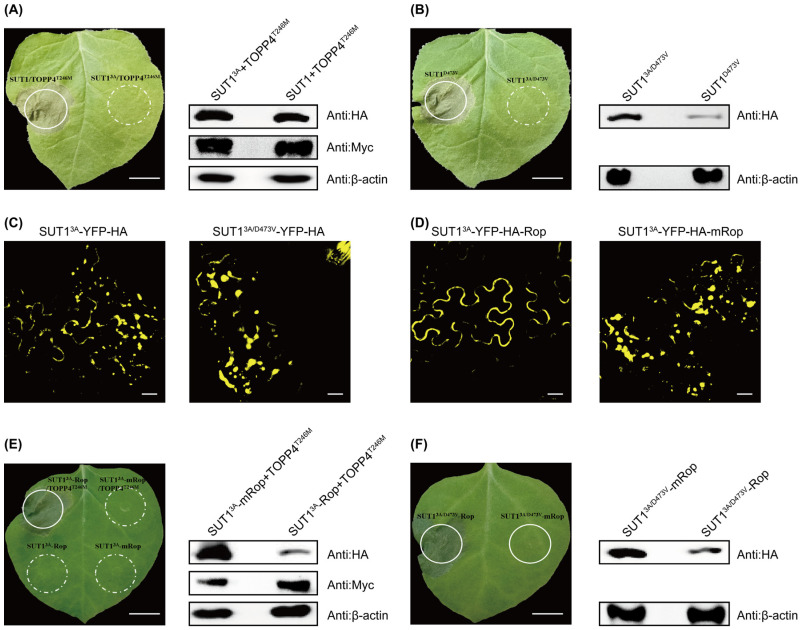
PM localization is crucial for the function of SUT1. (**A**) Mutations of Gly2, Cys4, and Ser6 residues to alanine (designated as 3A) abolish SUT1 activation; TOPP4^T246M^ cannot drive activation of SUT1^3A^. (Scale bar: 1 cm). (**B**) The cell death-inducing activity of SUT1^D473V^ was abolished by the 3A mutation. (Scale bar: 1 cm). (**C**) 3A mutation directly affects the cellular localization of SUT1 and SUT1^D473V^. (Scale bar: 20 μm). (**D**) The Rop tag can successfully anchor the otherwise cytoplasmic SUT1^3A^ to the PM. (Scale bar: 20 μm). (**E**) The SUT1 functions on the PM. (Scale bar: 1 cm). (**F**) Forcibly anchored SUT1^3A/D473V^ on the PM triggers cell death. (Scale bar: 1 cm). All data are representative of three independent experiments with consistent results.

Several residues at the N-terminus, such as Gly2, Cys4, and Ser6, contribute to the PM localization of SUT1 (also known as R5L1) [31]. A G2A/C4A/S6A mutation (named 3A thereafter) was introduced into SUT1 and SUT1^D473V^ to evaluate the impact of these residues on SUT1. The results showed that TOPP4^T246M^ cannot drive activation of SUT1^3A^ (Figure 3A), and SUT1^3A/D473V^ was deficient in inducing HR in *N. benthamiana* (Figure 3B), suggesting the importance of Gly2, Cys4, and Ser6 residues in the function of SUT1. The subcellular distribution of SUT1^3A^-YFP-HA and SUT1^3A/D473V^-YFP-HA were investigated. Unlike membrane-localized SUT1, SUT1^3A^ and SUT1^3A/D473V^ were found to exhibit punctate intracellular fluorescence in the leaves of *N. benthamiana* (Figure 3C). The results indicate that PM localization is required for SUT1 to induce HR.

The Rop tag can target proteins to the PM due to myristoylation and palmitoylation [32]. We fused the Rop tag to SUT1^3A^-YFP-HA and SUT1^3A/D473V^-YFP-HA, respectively. A mutant Rop (mRop) that could not be acylated was made as a negative control. The Rop tag, rather than mRop, restores predominant, but not exclusive, membrane localization (Figure 3D). We then co-expressed SUT1^3A^-Rop and TOPP4^T246M^ in *N. benthamiama*, and SUT1^3A^-Rop can be activated by TOPP4^T246M^ to cause HR (Figure 3E). In addition, SUT1^3A/D473V^-Rop, but not SUT1^3A/D473V^-mRop, displayed autoactivity in *N. benthamiana* (Figure 3F). The results further confirmed that the PM localization is necessary for SUT1 to induce HR.

### 3.4. SUT1 Function Is Affected by Mutations in Some Specific Residues

**Figure 4:**
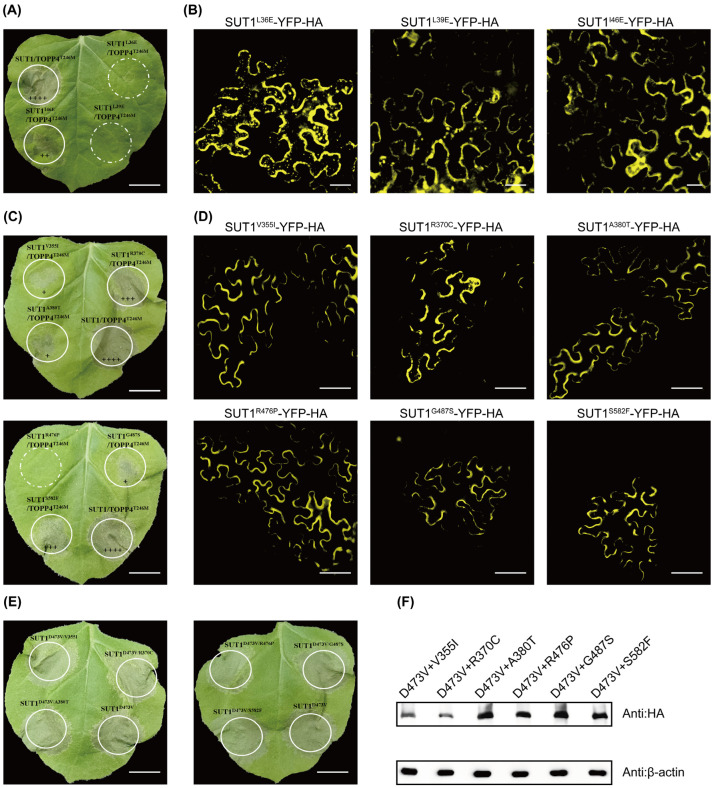
SUT1 function is affected by mutations in some specific residues. (**A**) Mutations of three hydrophobic residues (L36, L39, and I46) in the CC domain can compromise or abolish the function of SUT1. + indicates the severity of cell death. (Scale bar: 1 cm). (**B**) The functional deficiency of the SUT1 alleles in triggering HR is not due to the lack of protein expression. (Scale bar: 20 μm). (**C**) SUT1 mutants co-expressed with TOPP4^T246M^ in *N. benthamiana.* (Scale bar: 1 cm). (**D**) Observation of subcellular localization of SUT1 mutants. (Scale bar: 20 μm). (**E**) Cell-death phenotypes of YFP-HA-tagged indicated SUT1^D473V^ mutant versions. (Scale bar: 1 cm). (**F**) Immunoblotting confirms that all SUT1^D473V^ alleles were correctly expressed. All experiments were performed in triplicate; the obtained results are the mean of three independent experiments showing consistent results.

Some hydrophobic residues in the CC domain are important for MLA10 function and its CC dimerization [33], and these residues are conserved in SUT1, MLA10, and RPM1 (Figure S2). We tested these SUT1 mutants for their ability to induce HR in response to co-expression of TOPP4^T246M^, and found that they either partially or totally lost TOPP4^T246M^-dependent SUT1 activation (Figure 4A,B).

We also reconstructed six previously reported SUT1 loss-of-function alleles (sut1-1 to sut1-6) in *Arabidopsis* [25]. SUT1^V355I^, SUT1^R370C^, and SUT1^A380T^ are mutants close to the GLPL motif. SUT1^R476P^ and SUT1^G487S^ are mutants adjacent to the MHD motif, and SUT1^S582F^ is a mutant in the C-terminal LRR domain. When they were co-expressed with TOPP4^T246M^, these SUT1 mutants showed impaired abilities to cause HR in *N. benthamiana* (Figure 4C,D). However, we surprisingly found that none of the tested mutation sites obviously affect the autoactivity of SUT1^D473V^ in *cis* (Figure 4E,F). These results suggest these six mutations may maintain SUT1 in a low-activity but not inactive state.

## 4. Discussion

Our experiments show that TOPP4 mutants can activate SUT1, but rule out the possibility of activating SUT1 by monitoring the phosphorylation status of TOPP4 at the T246 site (Figure 1C). Yan et al. confirmed that SUT1 is unlikely to be a phosphorylation substrate of phosphatase TOPP4 [25]. We propose two possible hypotheses. First, the mutation at the T246 site may alter the spatial conformation of TOPP4, meaning that SUT1 is activated by directly sensing the conformational changes in TOPP4. Second, the T246 residue mutation might affect the function of TOPP4, preventing true guardee from undergoing dephosphorylation modification and indirectly activating SUT1.

Effector-independent activation of SUT1 mediated by TOPP4 alleles requires the P-loop within the SUT1 NBS domain (Figure 1C and Figure 2A). A widely accepted view regarding NLR activation is that binding to ligands promotes ATP/dATP exchange of ADP molecules in a resting state (bound to the NOD domain), leading to oligomerization of NLR [34,35,36]. However, the NOD domain of the tetrameric RPP1 resistosome binds ADP instead of ATP/dATP in the pentamer ZAR1 resistosome [35,37]. Our results are consistent with a model wherein SUT1 binds ATP/dATP when activated, since a loss-of-function SUT1 P-loop mutation not only blocks TOPP4 mutants-mediated SUT1 activation, but also abolishes the autoactivation of SUT1 MHD version (Figure 2A,D). In addition, the recognition of the γ-phosphate group of ATP in the ZAR1 resistosome and Apaf-1 apoptosome is facilitated by an arginine residue in the conserved ‘TT/SR’ motif [35,38], which is important for their activation and preserved as ‘TTR’ motif and R292 in SUT1 (Figure S2).

Activation of SUT1 can be driven by TOPP4^T246M^ (Figure 1A). The Gly2, Cys4, and Ser6 of SUT1 are crucial sites for its PM localization and function (Figure 3A–C). Both TOPP4 and TOPP4^T246M^ interact with SUT1 on the PM in *N. benthamiana* and with the CC domain of SUT1 in yeast [25]. Therefore, it is plausible to assume that the Gly2, Cys4, and Ser6 residues in the CC domain drive SUT1 to the PM, where SUT1 interacts with TOPP4 and TOPP4^T246M^. The fact that the N-terminal domains of the NLR mediate the interaction with guardee is reported for other plant NLRs [16,39,40].

Significantly, in this study, the wrinkled grayish leaf phenotype observed in *N. benthamiana* indicated that activated SUT1 triggers cell death; however, we did not directly demonstrate its classification as programmed cell death (PCD). The principal hallmark of PCD is not a change in PM permeability but specific chromatin remodeling [41]. Future work could employ DAPI or Hoechst dye to confirm cell death and, in parallel, show chromatin status for further analysis.

## 5. Conclusions

In this study, we found that co-expression of SUT1 with TOPP4 mutant versions, instead of wild-type TOPP4, produced robust cell death in *N. benthamiana*. The YFP-labeled SUT1 was localized on the plasma membrane (PM), and Gly2, Cys4, and Ser6 are crucial amino acid sites for its PM localization and function. Further dissection proclaimed that the function and localization of SUT1 are influenced by mutations in conserved specific residues.

## Data Availability

All data are available within this manuscript.

## Supplementary Materials

The following supporting information can be downloaded at: https://www.mdpi.com/article/10.3390/life16020227/s1, Figure S1: Single expression of YFP-HA-tagged SUT1 and Myc-tagged TOPP4 or TOPP4^T246M^ did not induce HR; Figure S2: Partial sequence alignment of RPM1, MLA10 and SUT1.
