# Supplementary material for: Dissection of Cell Death Induction by Arabidopsis thaliana CC-NBS-LRR Receptor SUT1 and Its Interacting Protein TOPP4 Mutant in Nicotiana benthamiana
Source: Life (Basel). 2026 Jan 29;16(2):227. doi: 10.3390/life16020227 (PMC12941724; doi:10.3390/life16020227)
Supplement: Supplementary file 1 [file life-16-00227-s001.zip › life-4118319-supplementary.pdf]

Supplementary Material

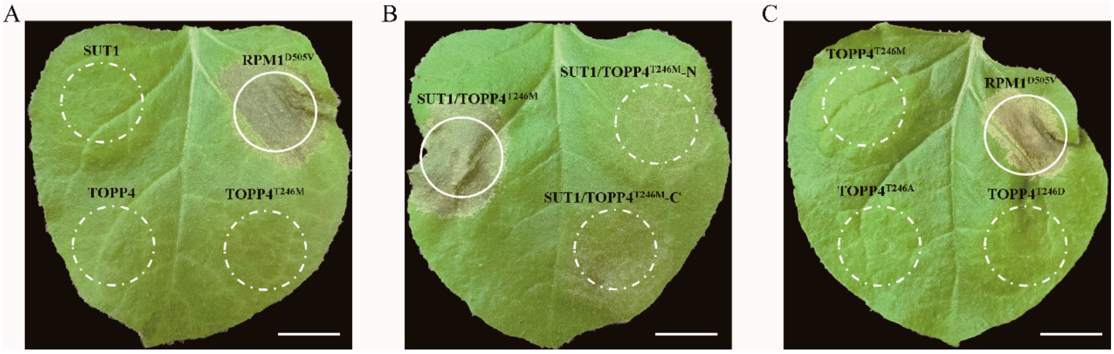

Figure S1 Single expression of YFP-HA-tagged SUT1 and Myc-tagged TOPP4 or TOPP4^{T246M} did not induce HR. (A) *35S::SUT1-YFP-HA* and *35S::TOPP4-Myc* or *35S::TOPP4^{T246M}* was transiently expressed in *N. benthamiana* by *Agrobacterium* infiltration (OD_{600}=0.4). *35S::RPM1^{D505V}-YFP-HA* was used as a positive control. Picture was photographed at 72 hpi. (Scale bar: 1 cm). (B) The full-length TOPP4^{T246M} mutant is necessary for activating SUT1. Full-length TOPP4:1-321 amino acid (aa); TOPP4^{T246M}-N:1-161aa; TOPP4^{T246M}-C:162-321aa. (Scale bar: 1 cm). (C) The *35S::TOPP4* mutants alone cannot induce cell death in *N. benthamiana* (OD_{600}=0.4). (Scale bar: 1 cm).

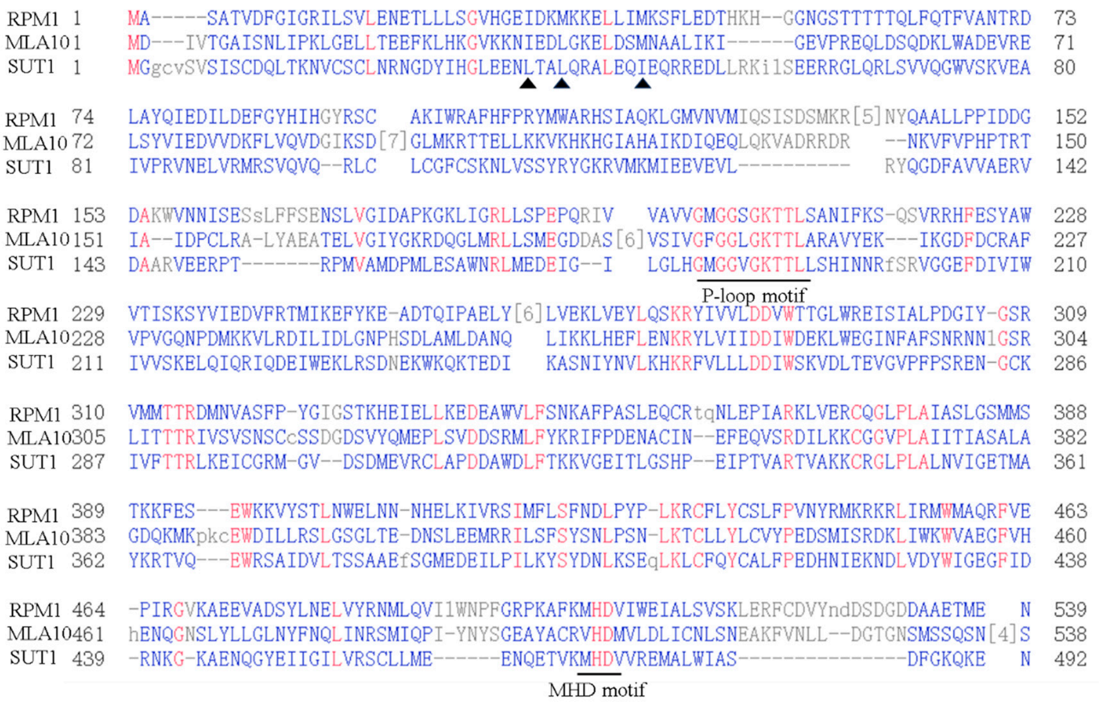

Figure S2 Partial sequence alignment of RPM1, MLA10 and SUT1. The positions of the P-loop and MHD motifs have been indicated by line segments. The black triangle indicates the three conserved residues in RPM1, MLA10 and SUT1 mentioned later.
